# Geophilosophical realness of risk: a case study in national housing authority resettlement sites in Albay, Philippines

**DOI:** 10.1007/s42452-021-04442-6

**Published:** 2021-03-24

**Authors:** Ana Marie R. Abante

**Affiliations:** Bicol University, Legazpi, Albay Philippines

**Keywords:** Risk assessment, Risk hotspot, Stability, Site selection, Risk governance

## Abstract

The *geophilosophical* realness of risk, as introduced in this study, is composed of the risk hotspot or cold spot information which are stored and sorted in hexagonal bins representing the host environment within the 25-km radius from the crater of the Mayon Volcano. The *z *scores measured from these hexagonal bins mimic the risk realness or risk reality phenomenon happening in Albay Province, Philippines. The objective of the study is to assess risk reality phenomena that generate risk knowledge originated from applying the seven metatheorems based on the Schoen Golden Triangle and the Fibonacci Golden Ratio. Risk assessment in this study uses the stability site selection criteria and hexagonal binning technique to store, sort, and process risk hotspot and coldspot information. This approach led to the disclosure of risk phenomenon on the 14 out of 25 resettlement sites (host environment) that remained at risk and continuously increasing the risk trend. When people are continuously allowed to occupy risk hotspots areas it hints at ineffective risk governance to neutralize the passively exposed population. This study concluded that the risk reality phenomena assessment opens new avenues for scientifically informed land use, nil exposure, and 0-risk policy in addition to the existing 0-casualty goal to get prepared with the right direction, decision and action to sensitively utilize the stable host environments aligned to improve risk governance.

## Introduction

The Philippines is the third most disaster-prone country in the world, according to the World Bank, and there is a low uptake of research and analytic thinking to inform local decision making on disaster risk management. According to the Bicolano historian Dr. Danilo Gerona the documentation on disaster risk reduction in the Province of Albay, Philippines can be found as early as 1814, when the Mayon Volcano erupted, disrupting people’s lives, and forcing them to look for other lands suitable for settlement and agriculture which are far from the volcano but near rivers and accessible to trade and communication. The Philippine Institute of Volcanology and Seismology (PHIVOLCS) is mandated to monitor volcanic related events specifically on gullies and barrancas within the Permanent Danger Zone. This institute together with the respective local government units which are normally affected by the volcano are alerted to be vigilant during periods of unrest and desist from entering the danger zones during eruption events. Residential buildings, roads, and other socioeconomic support structures continue to increase within the 6-km permanent and 8-km extended danger zones noted by the institute. As multiple hazard events naturally occur, the land morphology undergoes physical and environmental modifications, making people intrinsically vulnerable to the dangerous environmental changes. The evacuation-return behavior of farmers within a complete no-build-zone model (the Mayon 6-km Permanent Danger Zone of Albay), allowing barangay social and institutional facilities such as schools, barangay halls, and other development, is becoming the new norm before the COVID-19 pandemic and has contributed to uncontrolled sprawl within the permanent and extended danger zones of the tri-nodal spatial development in Legazpi City, Tabaco City, and Ligao City. The undesired developments in Albay can be presented and visualized by analyzing the host environment with OpenStreetMap spatial data as well as the continued construction of buildings along old railroads and rights-of-way near rivers that are prone to flash floods carrying lahar deposits in ArcGIS platform as a tool to assess risk [1–2].

The gap-bridged this study introduces is how to practically measure the risk quantities regarded as hotspots or coldspots (binned in hexagonal shaped host environment) to create risk knowledge on the geographical aspects of the space discovering where the hotspot information that are influenced by location-based risk reality phenomenon.

In the context of the risk reality suffered by the people in resettlement sites, this study focused on analyzing the borderline of risk and resiliency. This was made possible by analyzing the elements of risk relative to its geographic location. In this study, risk elements are construed by six variables, namely: single to multiple hazards, landscape vulnerability (condition of the host environment), exposure (geographic coordinates depicting the nearness or proximity of roads and building footprints to the danger zones), preparedness (dependent on utilization and conformity with the highest-best-land use or no-build zones policy directions), competency (limited income class of cities and municipalities in Albay), and coping capacity (also limited income class). The study also analyzed the categorical independent variable that can control the phenomena that is important in analyzing the risk reality suffered by the resettlement sites.

The study introduces the geophilosophical realness of risk and proposes a technique to find where the risk hotspot and coldspot information that brings insights about the stability or instability of resettlement sites’ as a host environment. This concerns environmental planners, researchers, decision-makers, risk reduction managers, and policymakers who need to measure risk by using binning techniques to visualize and analyze the spatial representations of risk elements that are stored and sorted in the hexagonal bins as host environments to improve risk governance.

This paper aims to show the risk reality suffered by the resettlement sites in Albay based on stability criteria to check if the sites (host environment) were selected, respectively, and responsively. To do this risk hotspot analysis was done to locate and assess risk reality phenomenon level of significance. The level of risk hotspot level of significance inquiry applied tessellated cells that characterized the risk realities suffered in the host environment. The researcher developed a Geographic Information (GIS) model that will demonstrate risk reality phenomenon is measurable. The specific objective is to generate risk reality information to generate risk knowledge based on stability criteria, risk elements and trends, and land use that can advance preparedness or meaningful risk reduction in the host environment (resettlement sites) or putting risk reduction and management where it needs to be.

The geoplilosophical realness of risk suffered by the resettlement sites can provide an important framework for selecting a stable host environment for settlement or resettlement site, thus significant in restoring stability and keeping a sustainable growth for cities and municipalities in the province of Albay, Philippines.

While it is difficult to examine the consequences of not employing proper selection criteria for the host environment of resettlement sites or unknown metes and bounds of risk and resilience risk measurement is hard. Risk measurement is seen significant in analyzing conforming and non-conforming to suitably select highest-best-land uses. Bearable or tolerable risk information is meaningful in analyzing what are the adaptable (allowable) measures to counter risk as climate changes. Risk measurement directs the actions and decisions to restore stability and sustain developments. Seeing that the 0-casualty goal of Provincial Government of the Albay is limited to perfecting the communication protocols to evacuate and return (institutionalized) practices in Albay seemingly becomes a new normal before the COVID-19 pandemic. A new norm after this pandemic hint to combine the natural and man-made hazards and risk measurement concepts and techniques are foreseen needs in environmental planning to get prepared in advance.

This paper will try to open the minds of planners, engineers, decision-makers, policymakers, developers, and researchers to undertake a scientifically informed risk reduction originated from the geophilosophical risk realities perspective and metatheorems to correctively and sensitively take actions that advocate a forward-thinking risk governance. A do-nothing scenario gets the beneficiaries of government housing projects to stay unprepared from the worsening risk reality which can prolong recovery that implies an unstable and unmanageable different resettlement sites. Furthermore, this paper also presents the risk knowledge created by partitioning the DRRM cycle based on the Fibonacci Circle that can possibly adjust or define where the prevention, mitigation, preparedness, response, and recovery starts and ends to advance preparedness [1, 16]. These partitions also hint at analyzing common mistakes in assessing risk reality phenomena. Unlocking these well-defined partitions suggest a better way to rethink how to build-back-better or restore the vulnerable host environment (resettlement sites as examples) [1–2, 15–16].

## Framework

### Literature review

Resettlement is the act of settling displaced or exposed residents in another built environment. The current literature on resettlement focuses more on qualitative research that describes the issues concerning the land acquisition relative to the displace or exposed people settling to another built environment or social stability risk within the host environment and its linkages with resettlement financing, project lifecycles, paucity of outcomes experienced by displaced populations in relation to their threats to livelihood and safety or hazard susceptibility of resettlement sites as host environment. Some studies focus on economic losses, costs of undertaking resettlement work, valuation of land, housing, and property value, and emergency cost, and local adaptation to climate change. Few studies focused on assessing the evacuation and return practices or resettlement projects that were institutionalized under strict communication protocol under the 0-casualty goal in the Provincial Government of Albay. These studies matter but there are no clear guidelines that mandate how to measure risk and its elements to weigh the needs to restore stability to properly allocate land to its highest-best-land use as a strategy to mainstream risk measurements and adaptation of bearable risk quantities into land use plans or local resiliency plans. The current criteria for site suitability analysis to realize physical and environmental balance is vague in the guidelines to prepare land use plans and zoning. There are several studies that only highlight the vulnerability assessment on the host environment or people impacted by natural calamities and hazards. The current related literature and studies do not discuss analyzing where the risk hotspots are located, risk remainders from the past disasters or extraneous errors in risk modeling in the context of space quantity or to observe the borderline that distinguish risk from resilience or vice versa. There are quantitative-based studies but limited to mathematical models that fundamentally represent the risk or resilience related phenomena. But to make a fabric of risk reality, there is no quantitative research that applies metatheorems on risk space quantity or stability based on Fibonacci spirals that are present in a space where risk quantity is bearable or disaster as space that is beyond measurable to support meaningful risk governance and beautiful life that lies ahead if we overcome all disaster risk related challenges. Albert Einstein once said the definition of insanity is doing the same thing over and over again and expecting the same result.

In the words of Owen et al. [3] the population displacement caused by resource development projects is a difficult phenomenon to deny responsibility. Their study disclosed the essential relationship between the industrial projects (land development) and the host (geographical location) environment which influences the market economics. According to them, when developers fail to account for, or “own” the costs of undertaking resettlement work, a large unmeasured portion of this cost is often transferred into the external (extrinsic landscape condition) environment. Their concept model explained the linkages between resettlement financing, project lifecycles, and the paucity of outcomes experienced by displaced populations [3].

The work of Gong et al (2020a, b) explored the livelihood problems following development-induced displacement and resettlement [4]. Their livelihood resilience inferred measurement model measured the livelihood resilience of displaced population and proved that there is a relationship between the income structure from the original settlement to the resettlement site and the distance (access) of agriculturally based livelihood. The livelihood resilience knowledge put forward the preparations (risk reduction) needs that guided the governments in resettling the population displacement. The livelihood resilience measurement concept of Gong et al (2020a, b aimed to measure resilience based on Hooke’s law to test the effectiveness using correlation analysis to calculate livelihood resilience scores. The samples were based on land (geographical location) ownership, housing, and property value, and emergency cost are the most significant of these factors to help the government to monitor and regulate projects aiming at building livelihood resilience [5].

As said by Hofmann [6] there is no agreed upon definition of the concept of “resilience”, even though the term is increasingly used in the research on effects of environmental changes on natural and social systems. His concept of resilience is a relative one, i.e., it depends on the more primitive concepts of the entity: resilient, disturbance of the entity, situation before and the situation after the disturbance occurred, and the similarity criteria to compare the situation before the disturbance occurred to the situation thereafter [6] In the words of Uy et al. [7], local adaptation to climate change is essential for vulnerable communities faced with increasing threats to livelihood and safety. Their paper seeks to understand the micro-level enabling conditions for climate change adaptation through a livelihood lens in Albay. Their findings on the micro-level variations in the villages suggests that the understanding of local conditions is indispensable in planning and formulation of appropriate adaptation strategies and actions at local level [7].

The paper of Peng et al. [8] focused on modeling the social stability risk of land acquisition and resettlement using fuzzy comprehensive evaluation methods to assess the social impact based on international and China evaluation standards. Based on a dual requirement, the authors reviewed the influencing factors, sources, evaluation methods, existing problems and suggestions of the major decision-making body, policy, reform, and construction of a hydropower station to maintain economic and social stability. Based on the social stability risk assessment highlighting the similarities and differences between evaluation standards, the social stability risk of land acquisition and resettlement knowledge showed the complexities to meet the social risk management requirements to keep stability in the host (geographical location) environment [8].

According to Aven and Flage [9], the need for risk knowledge has risen recently, and methods and approaches for risk assessment are to be seen. It supports incorporating the knowledge into a systematic, rigorous, and transparent framework that generates knowledge about risk probabilities and measurement. They said that risk is conditional on the knowledge is typically based on data and it takes the form of justified beliefs (often stated as assumptions in risk models and characterization) while probability is a judgment of uncertainty [9].

As stated in the book of Bosher and Chmutina [10] the impacts of climate change can affect the built environment directly and indirectly which could lead to the destruction of physical assets and property, and widespread displacement of people. They defined vulnerability as the characteristics and circumstances of a community, system, or asset that make it susceptible to damaging effects. The consequences of the direct impacts are correlated with demographic, economic, and political stressors that increase intrinsic vulnerability (e.g., poverty, political instability). According to them, urbanization and the risk factors induced by climate and other hazards and threats create a diverse range of vulnerabilities (often discussed in the context of urbanization) referring to the exposure of inhabitants and systems to the disturbances, such as natural hazards, economic crises or political unrests, exacerbated by population dynamics, informal settlements and inappropriate governance and planning. They quoted Bene (2013), on the three main factors that multiply the risks generated by urbanization: Geographical location relative to extreme weather events and human-induced threats, dependence on the complex systems that are vulnerable to various threats and hazards, and the level of resilience and the governance of resilience in which the government capacities are often unable to regulate development which leads to increase in vulnerabilities [10].

In the words of Daep et al. (2021), the Albay Province in the Philippines successfully achieved a 0-casualty goal for the past two decades under a strict communication protocol [11]. Be that as it may, Espinas [12] said that multiple hazards impacting Albay are primarily influenced by its location and geographic landscape. She stressed that geography and environmental phenomena should not hinder development. She also said that Albayanos can choose to overcome and conquer the challenge, but more work awaits before safe development is realized [12]. Authors Cuevas et al. (2015) said that mainstreaming climate change adaptation (CCA) into development plans is still a new approach in adaptation and thus there is limited information on how to operationalize it on-ground. They tried to examine the gap by investigating the challenges in mainstreaming CCA into the local land use plans in the province of Albay, Philippines. Their findings suggested 20 quantitative “mainstreaming indicators” categorized into: information, institutional (fragmented laws and regulations; overlapping policy requirements; and the lack of guidelines for mainstreaming CCA into the local land use plans), and resource capacities of systems (influenced by “leadership” indicator where the champion effectively led the CCA efforts because the existing institutional mechanisms at the provincial level which relatively influenced the behavior of Albayanos in production of collective action towards CCA [13] Espinas [12] and Cuevas et al. (2015) recognized the importance of location and risk information in the safe developmental policy direction, decision making and action to reduce risk or strengthen CCA [11–13].

Abante (2020a, b) mentioned Birkmann (2006, 2013) theory on measuring vulnerability to promote disaster-resilient societies. His theory stressed the need for a paradigm shift from qualification and analysis of the hazard to the identification, assessment, and ranking of vulnerabilities, underlining the importance of measuring vulnerability and developing (extrinsic) indicators to reduce risks and the (intrinsic) vulnerability of the societies at risk. According to him the ability to measure vulnerability is increasingly being a key step towards effective risk reduction and the promotion of a culture of disaster resilience. According to him his theory of measuring the risk and its remainders as a challenge with a notion that discloses the idea of measuring the ‘unmeasurable’ risk, referring to the challenges and difficulties in deriving appropriate methodologies, indicators, and site selection criteria to identify, measure, and assess vulnerabilities of societies at risk [1]. Despite the significance of site selection few academic studies have attempted to understand the site selection process. They stressed that understanding the site selection process can stimulate important research issues, formulated site selection schema, conceptual model of the site selection process and put forward several research propositions. They said that research in this area has the potential to contribute substantially to improve decision making and better understanding of reducing expenditures which benefits both the host (analogous to the local government unit) and guest (analogous to the resettlement project beneficiaries) [1, 11–13].

Barua et al (2020) mentioned Abante and Abante (2018) on the importance of processes to mainstream risk reduction and management into sensitive land use planning in recent years. According to them the combination of analytical and participatory approaches which has been applied to ensure applicability as well as acceptability can guide policymakers to understand the importance and application of risk sensitive land use planning [14]. Authors Abante and Abante (2018) [15] said that there is a need to understand the (insufficient) preparedness as an element of risk relative to the state of preparedness as one of the circumspectial phases understood as partitions of DRRM that are becoming crucial in risk reduction to meet the Sendai Framework. Likewise, Abante (2018) [16] also said that changing landscape conditions of the host environment and preparedness insufficiency can worsen the coping capacity of the local government as well as the displaced people which may cause problems in meeting the Sendai Framework.

Abante (2020a, b) cited Woodward (2005, 2013) who quoted Gilles Deleuze (1925–1995) and Félix Guattari (1930–1992) on the term ‘*Geophilosophy’* they used to analyze adaptation of geographic concepts that emphasizes proximity, contingency, interdisciplinarity, and bottom‐up modes. For them, geophilosophy has also influenced numerous theoretical and practical developments in geography, such as nonrepresentational, affect, and assemblage theories. She also quoted Nicola Masciandaro (2010) on his definition of the term *geophilosopher* as “one who philosophically experiences rather than frees the earth, who passes through and remains with it”. Masciandaro emphasized *Geophilosophical* experience that entails facing, more and more deeply, the fact of the earth as the place of philosophy, and more profoundly, experiencing the wrath as facticity itself, the site thought’s passage to the absolute. The researcher’s curious mind and strong analytical skills are fulfilling to share her *Geophilosophical* thoughts [1]. Abante (2020a, b) also mentioned King (2020) on citing the Christaller’s Theory on Central Space on the role of urban places as service centers which determines the character of the major economic activities carried on within the city, town, or village [17]. Abante (2020a, b) agrees with King (2020) on the importance of attempting to develop a theory of central places that emphasizes the economic interrelationships between them and the rural areas [1, 17]. As presented in the Abante (2020a, b) risk reality phenomenon concept, based on the Fibonacci Golden Ratio and Schoen Golden Triangle theories hinted the 0-Risk (resilience measurement) is significant when exposure is nil or lacking. Resiliency is measured where the stability changes and is located [1, 5] To determine the location of risk, the Abante (2020a, b) practically used Christaller’s Theory to store the elements of risk to analyze the patterns of risk and resiliency (based on sensitivity and conformity to the higher-based-land use to measure preparedness sufficiency as one of the six elements of risk) which mimicked the risk reality phenomenon in Albay [1–2, 17].

The Geophilosophical realness perspective based on the seven *Metatheorems* objects based on the Fibonacci Golden Ratio and Schoen Golden Triangle contributes to the discovery of the criteria of balance metatheory where stability is constantly pivoting at the Vertex of Resiliency. The first *Metatheorem* object on the Risk Reality Triangle analytical model as shown in Fig. 2 proved that when risk reality quantity is greater than one unit of risk quantity it connotes that risk hotspot information is true. On the contrary, when the risk reality phenomenon is less than one unit of risk quantity it is regarded as cold spots. Coldspot is also regarded as nil or 0-risk quantity when the independent variable on exposure coincidence is unlikely. The second Metatheorem object on the Receptiveness–Responsiveness–Stability Triangle analytical model asserts that the isosceles triangle asymptotic (Receptiveness and Responsiveness quantity) segments control the base from increasing. The asymptotic segments represent the receptiveness knowledge that is correlated to safe space (the function of multiple hazards and passive exposure) while the responsive knowledge is correlated to a comfortable environment (a function of passive exposure and landscape vulnerability of the host environment) and accessibility knowledge. The third Metatheorem object on the Stability Quantity based on the Fibonacci Golden Ratio and Schoen Golden Triangle regarded as the base of the smaller isosceles triangle ABC as shown in Fig. 2 proved that the equation of the Intergovernmental Panel on Climate Change (IPCC) of the United Nations as the body for assessing the science related to climate change was interpreted as = , the segment of the isosceles triangle. The fourth *Metatheorem* object on the Unreceptive-Unresponsive-Fuzzy Reality Quantity analytical model, regarded as where the larger isosceles proved that the geometric center is positioned at (0,0) with a risk quantity of 0.008. It reckons the stability relative to the asymptotic line segments that are also applicable to the series of isosceles triangles as shown in Fig. 3. The risk reality phenomenon symbolized as Phi φ is based on the Fibonacci Golden Ratio and Schoen Golden Triangles, mathematically written as Risk Reality Phenomenon Phi *φ* = *R*^2 + (2Cosine72°*R*)/AB-(2Cosine72°)^2/AB or Phi *φ* = *R*^2 + 0.00494427*R* − 0.00305572. The fifth *Metatheorem* object on the Vertices of Resiliency analytical model proved that the vertices that are based on Schoen Golden Triangles Theory are the vertices of resiliency that describes the metatheory on the vertices that are constantly orbiting the spiral curve geometrical object. The risk reality phenomenon embodied by a spiral curve originated from the 25 Series of Isosceles Triangles objects whose vertices were reckoned from the Geometric Center Vertex of the first triangle. It proved the metatheory on the Vertex of Resiliency becomes an insignificant risk measurement as it approaches a near-zero risk. The sixth *Metatheorem* object on the Risk Reality Spiral analytical model characterizes where the state of balance (stability) as metatheory that rests in the risk reality phenomenon is curved. It disclosed where the maximum risk reality quantity is located. The inflection chart as shown in Fig. 4 revealed the risk reality phenomenon Phi described by the spiral is described as a risk quantity with 47.74 *z *score (hotspots with a 90% level of significance). Similarly, the risk reality phenomenon Phi with a 64 *z* score is regarded as a 95% level of significance. The 99% level of significance risk reality phenomenon can bring catastrophe, environmental damages, or economic activity disruption. If *z *scores reach 125 unit-level it practically implies that risk reality phenomenon becomes unmeasurable or fuzzy. This fuzzy risk reality phenomenon metatheory is described nearing the spiral tail as shown in 23rd and 24th triangles as shown in Fig. 3 and Table 1. The last *Metatheorem* object on the Fuzzy Risk Reality Circle analytical revealed that the risk reality phenomenon which is depicted by a circle originated from continuing diagramming. It disclosed the correlation of the geometric center and the tail of the circling risk reality phenomenon. The risk reality phenomenon circle is correlated and fitted to the DRRM cycle and stages in which it splits the cycle and stages into 5 parts of the risk reality phenomenon [1] (Fig. 9).

The first part of the risk reality phenomenon is where the prevention arc-length as shown in Fig. 6 should be measured to assess the risk reality phenomenon at the starting phase. Prevention measurement starts at the State of Alertness and ends at the State of Emergency. Similarly, the mitigation measurement starts where the prevention impact is drawn and ends at the State of Calamity. Also, preparedness measurement starts where the mitigation assessment stops. This knowledge on prevention, mitigation, and preparedness measurement (drawn side by side the risk reality phenomenon spiral curve) practically implies that shorter prevention and mitigation arc measurements will be transformed into preparedness needs meaning the preparedness arc will be prolonged. A prolonged preparation implies instability as it will mess up the risk reality triangle analytical model. It also implies that insufficient preparedness will affect the response phase as it could lead to longer response and emergency operations every time a natural calamity or disaster event happens. To complete a risk reality phenomenon assessment, the recovery arc measurement must be assessed where the response phase stops and ends where the prevention starts [1].

#### Geophilosophical realness of risk concept

**Fig. 1:**
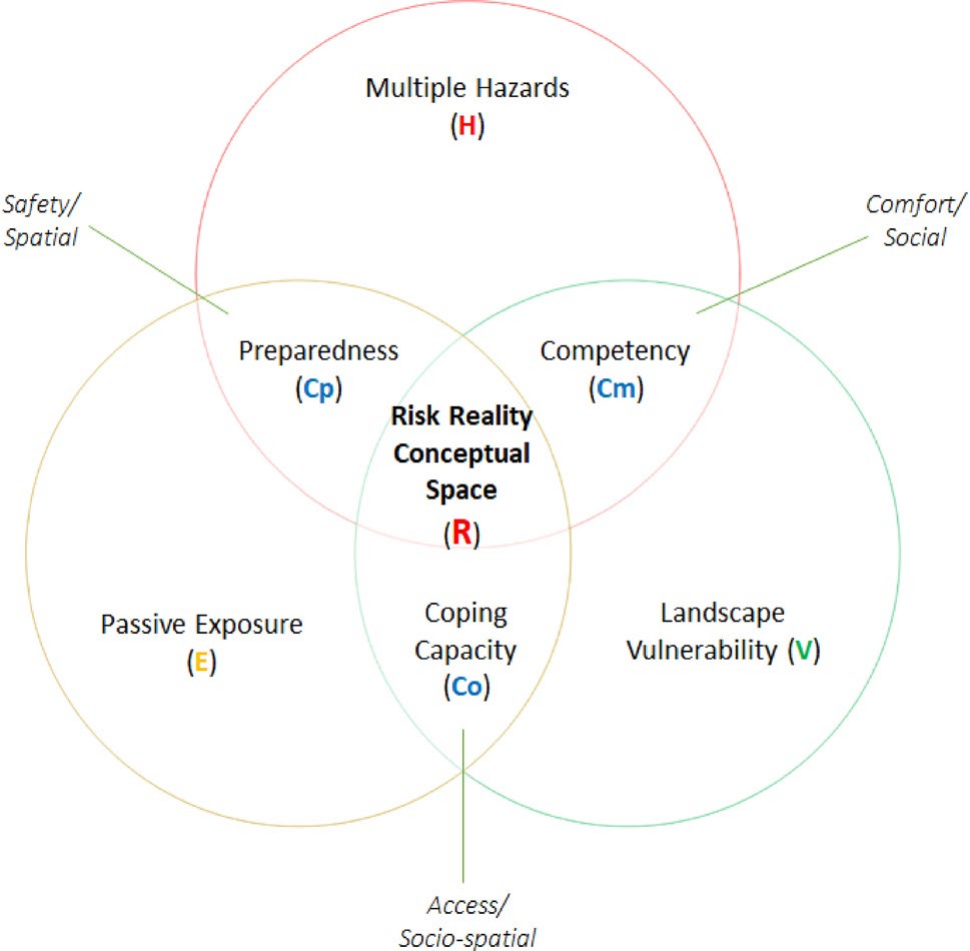
Risk reality conceptual model [1, 2]

This risk reality phenomenon concept was based on Gärdenfors ideas where the semantics stirred up reasoning to quantify the risk reality quantity with six variables of risk reality as shown in Fig. 1. Multiple Hazards (H) are natural features of disaster risk. It symbolizes the combined scores of multiple hazards in Albay. Multiple hazards denote the likelihood of events and aftermath within a specified period in each zone or geographical area at risk or are prone to potentially damaging natural phenomena. The importance of analyzing the effects of hazards or extreme weather events relies on the level of significance of vulnerability and exposure to weigh up adaptive capacity. Landscape Vulnerability (V) is regarded as the landform condition of the host environment called the landscape vulnerability is a landscape vulnerability variable of risk. It symbolizes the combined scores that characterize the condition of a piece of geographically enclosed land that is open to alteration (physical changes in the host environment) caused by floods, bank erosion, and other hydrological-related hazards that may occur when river discharge exceeds the channel’s volume, causing the river to overflow onto the downstream alluvial flats and the coastal areas resulting in different states of discomfort, security, worries, distress, angst, and more. Exposure (E) is regarded as the geographical location or static position of dwelling structures or intangible human assets that may be in hazard-prone areas likely to experience hazard events of different magnitudes. Preparedness (Cp) is regarded as an element of the overall capability of the government to fully recover the disaster risk and its residuals to prevent and mitigate to advance preparedness to keep a stable and sustainable development. It is correlated to the stability selection parameters that convey receptive action of the government to reduce risks. Competency (Cm) is regarded as responsive action that features one of the elements of the overall capability of the government to fully recover the disaster risk and its residuals to select and relocate displaced or vulnerable people to a comfortable environment and address the basic social needs of the target housing beneficiaries to be resettled. It is a variable that deals with responsive risk reduction that features the comfort element of stability that connects the people to the place of work and social support facilities. Coping capacity (Co) is also regarded as responsive action that features one of the elements of the overall capability of the government to eliminate if not reduce risk by moving the displaced people or extremely exposed settlements taking responsive actions taking consideration of their coping mechanism away from source of income or livelihood [1].

The Y-junction of Gärdenfors (2004) inspired conceptual model is attributed to three (3) logical paired variables, where risk reality is expressed as Risk function (multiple hazards, landscape vulnerability (landform of the host environment), passive exposure, preparedness sufficiency, competency and coping capacity of the government); H multiple hazards present in Albay; V landscape vulnerability within the foot slopes of Mayon Volcano; E passive exposure of road and building location information; Cp preparedness (tied with safe conceptual space and stability based on the receptive risk reduction action); Cm competency (tied with comfortable host environ and stability based on responsive risk reduction actions); and Co coping capacity (tied with receptive and responsive provision of access to host environment that fairly considering the competency and coping mechanism of people to be resettled ore relocated), mathematically written as

### Risk reality contextualization

In this study, risk reality measurement refers to the risk assessment results where the function (risk reality) ~ multiple hazards, landscape vulnerability of the host environment, passive exposure, preparedness, competency, and coping capacity. Risk reality phenomenon refers to the state of being at risk based on the Fibonacci Golden Ratio and Schoen Golden Triangle theories that originated from the vertices of resiliency metatheory that are measured starting from the geometric center. Risk reality phenomenon refers to the state of being at risk, wherein the state of actuality or the existing situation is objectively lucid in the resettlement host environment that are binned in hexagonal-shaped polygons in the ArcGIS platform.

#### Risk reality curve geometric center

**Fig. 2:**
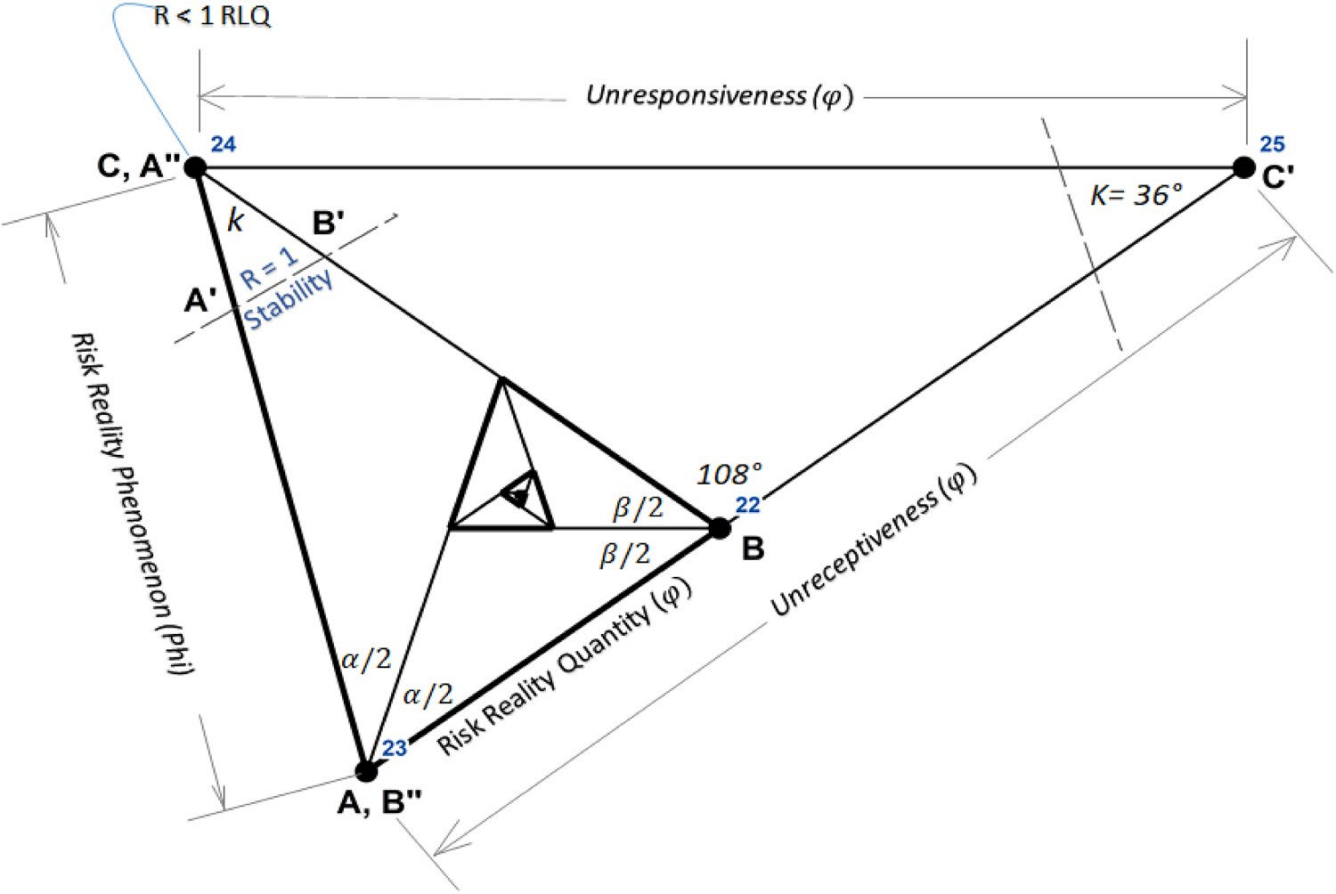
Risk reality phenomenon isosceles Δ [1, 2]

In this study, the conceptualization of the geometric center metatheory was based on the Fibonacci Golden Ratio and Schoen Golden Triangle. The geometric center as shown in Table 1 is where the risk reality reckoned with 0.008 risk quantity which implies resiliency. Figures 2 and 4. show that the geometric center is positioned at plane coordinates × 0 and y0 assumed to be the first vertex of the isosceles triangle ∆1 → lowest risk reality phenomenon that is measured at 0.008 risk or near 0-risk measurement define the resiliency measurement [1].

**Table 1:** Vertices of the resilience index grounded by the golden ratio and triangle

Series of resiliency vertices				Risk reality series of isosceles triangle based on Schoen golden Δ			Fuzzy reality based on fibonacci golden ratio (Phi)	Spiral risk trend (Arc Length)	Spiral risk trend conceptual space (RLQ Unit)	Remarks
Series of Δ	Vertex of resiliency (Angle $k=36^\circ and$ 108 ° rotation)	Vertex X	Vertex Y	Receptiveness (Segment AC)	Responsiveness (Segment BC)	Risk Reality (Segment AB) RRT Base (Phi)				
1	Vertex 1 of Δ 1	−0.004	0.013	0.013	0.008	0.008	−0.003	0.009		Geometric Center
2	Vertex 2 (When AB = AC = BC of Δ2)	0.013	0.000	0.022	0.013	0.013	−0.003	0.018	0.000	
3	Vertex 3 (When AB = AC = BC of Δ3)	−0.015	−0.021	0.035	0.022	0.022	−0.002	0.023	0.000	
4	Vertex 4 (When AB = AC = BC of Δ4)	−0.032	0.033	0.057	0.035	0.035	−0.002	0.046	0.000	
5	Vertex 5 (When AB = AC = BC of Δ5)	0.059	0.033	0.092	0.057	0.057	0.000	0.060	0.001	
6	Vertex 6 (When AB = AC = BC of Δ6)	0.013	−0.108	0.148	0.092	0.092	0.006	0.121	0.004	
7	Vertex 7 (When AB = AC = BC of Δ7)	−0.181	0.033	0.240	0.148	0.148	0.020	0.158	0.010	
8	Vertex 8 (When AB = AC = BC of Δ8)	0.133	0.261	0.388	0.240	0.240	0.056	0.317	0.029	
9	Vertex 9 (When AB = AC = BC of Δ9)	0.327	−0.336	0.628	0.388	0.388	0.150	0.415	0.067	
10	Vertex 10 (When AB = AC = BC of Δ10)	−0.689	−0.336	1.016	0.628	0.628	0.395	0.830	0.268	
11	Vertex 11 (When AB = AC = BC of Δ11)	−0.181	1.228	1.644	1.016	1.016	1.035	1.086	0.798	
12	Vertex 12 (When AB = AC = BC of Δ12)	1.972	−0.336	2.661	1.644	1.644	2.709	2.173	1.458	
13	Vertex 13 (When AB = AC = BC of Δ13)	−1.511	−2.866	4.305	2.661	2.661	7.090	2.844	8.643	
14	Vertex 14 (When AB = AC = BC of Δ14)	−3.664	3.759	6.966	4.305	4.305	18.553	5.688	12.925	
15	Vertex 15 (When AB = AC = BC of Δ15)	7.607	3.759	11.271	6.966	6.966	48.556	7.446	53.627	
16	Vertex 16 (When AB = AC = BC of Δ16)	1.972	−13.586	18.237	11.271	11.271	127.093	14.893	165.331	
17	Vertex 17 (When AB = AC = BC of Δ17)	−21.901	3.759	29.508	18.237	18.237	332.682	19.495	436.107	
18	Vertex 18 (When AB = AC = BC of Δ18)	16.726	31.823	47.746	29.508	29.508	870.889	38.990	1,002.238	
19	Vertex 19 (When AB = AC = BC of Δ19)	40.599	−41.650	77.254	47.746	47.746	2,279.891	51.038	4,098.835	
20	Vertex 20 (When AB = AC = BC of Δ20)	−84.401	−41.650	125.000	77.254	77.254	5,968.605	102.077	12,041.583	
21	Vertex 21 (When AB = AC = BC of Δ21)	−21.901	150.705	202.254	125.000	125.000	15,625.615	133.620	21,989.246	Vertex C
22	Vertex 22 (When AB = AC = BC of ΔABC	242.853	−41.650	327.254	202.254	202.254	40,907.770	267.240	130,954.125	Vertex *C*′
23	Vertex 23 (When AB = AC = BC of Δ ACC′	−185.528	−352.888	800.216	327.254	327.254	107,096.924	349.821	166,550.717	Vertex C″ (Spiral Tail/Spiral 23)
24	Vertex 24 (When AB = AC = BC of ΔCC′C″	−392.639	800.216	529.508	800.216	800.216	640,350.241	699.643	471,480.450	Vertex C’’’
25	Vertex C″	878.346	1270.985	800.216	529.508	529.508	280,381.810	855.399	646,888.220	Vertex “”
26			1270.985	529.508	529.508	800.216	640,349.600	1,639.514		
27			1270.985	529.508	529.508			1,782.081		Spiral 25

Author Contribution: Abante AMR (2020)

#### Risk reality phenomenon phi metatheorems

**Fig. 3:**
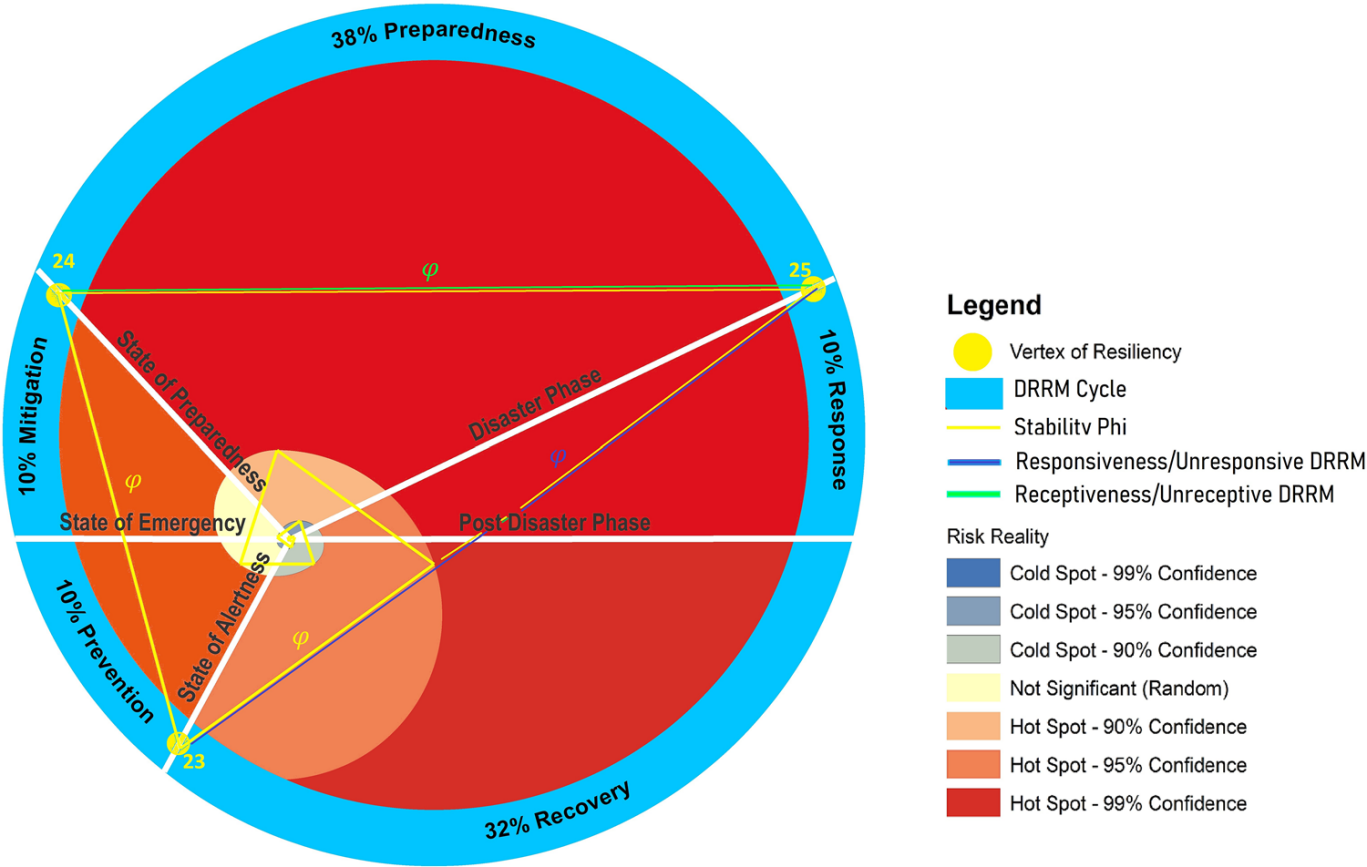
Risk reality circle grounded by Fibonacci golden ratio and Schoen golden triangle [1, 2]

The risk reality phenomenon Phi blueprint starts from 0-risk (resiliency) measurement and ends at 125 risk quantity defining the risk reality spiral curve. When R exceeds the 125-risk measurement (upper limit) it defies the risk reality phenomenon Phi blueprint which implies a new beginning is progressing from one sensitive phase to a more intense risk level. It can be mathematically written as Risk Reality Phenomenon Phi → 125 ≠ Fuzzy Risk Reality Phenomenon of new beginning shown in Fig. 3. This study proved that the Receptive-Responsive-Stability Isosceles Triangle ∆ ≡ with the Risk Reality Phenomenon Isosceles ∆ based on the Fibonacci Golden Ratio and Schoen Golden Triangle, where: C → Vertex of Resiliency → Angle kappa → 36° and AC → BC are asymptotic quantity segments with angle Alpha → *α* → angle Beta → *β *→ 72° and the *φ* upper limit → 125 and *φ* lower limit → 1 segment unit is regarded for the risk-reality → hotspot. Therefore, *φ > 125* represents the hotspot → Risk Fuzzy Reality, and *φ < *1 segment unit quantity represents resiliency or risk cold spots [1].

By diagramming a series of risk reality phenomenon triangles, it turned out that their vertices follow the angles: 36°, 72°, 108°, 144°, 180°, 216°, 252°, 288°, 304°, and 324°. The first ten risk reality phenomenon series of triangles as shown in Table 1 describe the resiliency metatheory because it measures less than one unit of risk quantity. The eleventh risk reality phenomenon triangle is assumed to be the stable state because its quantity nearing one unit of risk reality phenomenon, thus the asymptotic segments which define the receptiveness and responsiveness measurement, this smaller triangle (shown in Fig. 20 is where the resiliency measurement originates. Similarly, the bigger triangle symbolized by black lines as shown in Fig. 2 also expresses the Risk Reality Quantity Segment that is analogous to Risk Reality Phenomenon.

The *metatheoretical* presentation of stability and/or asymptotic line segments of the series of isosceles Δ*S* is regarded as continuous connectivity of *φ* when segments of the Δ*S* → base of the Δ represent the stability segment quantity → asymptotic segments representing the receptiveness and/or responsiveness metatheory → 1 → 1:1 ratio → when 125 < *φ*  ≥ 1 segment unit → it represents risk reality phenomenon metatheory. Thus, Phi > * φ* 125 risk quantity is regarded as the ‘fuzzy risk reality phenomenon or new beginning’ knowledge [1].

Analyzing the stability metatheory → where Phi *φ* → 1:1 ratio → regarded as receptiveness–responsiveness proportion → 1 risk quantity to the risk reality phenomenon $$\begin{gathered} \varphi \to \frac{125 < \varphi \ge 1 }{{125 < \varphi \ge 1 }} \hfill \\ \hfill \\ \end{gathered}$$ ratio that defines the Unreceptiveness-Unresponsiveness Proportion. The Risk Reality Phenomenon Phi *φ* originates from the *metatheorem* for Phi $\varphi \to \frac{\rm Risk Hotspot (Upper Limit)}{{\rm Risk Fuzzy Reality} (125/2{\rm Cosine}72^\circ )}\to \frac{\rm {Risk Hotspot} \left({\rm Upper Limit}\right)+ {\rm Risk Fuzzy Reality} ({\rm Risk Hotspot Upper} Limit/2Cosine72^\circ )}{{\rm Risk Hotspot} (Upper Limit)}\to$

$$\frac{{\rm Risk Hotspot}\left(Upper Limit\right)}{{\rm Risk Fuzzy Reality} \left(\frac{125}{2Cosine72}^\circ \right)}\to \frac{\mathrm{Risk Hotspot} \left(\mathrm{Upper Limit}\right)+\mathrm{Risk Fuzzy Reality}(\mathrm{Risk Hotspot Upper Limit}/2\mathrm{Cosine}72^\circ )}{\mathrm{Risk} \mathrm{Hotspot}(\mathrm{Upper} \mathrm{Limit})}$$

$\to \frac{125}{R (125/2\mathrm{Cosine}72^\circ )}\to \frac{125 + R(125/2\mathrm{Cosine}72^\circ )}{125}$ $\to$ $\frac{2\mathrm{Cosine} 72^\circ }{R}\to 1+ \frac{125R}{ 2\mathrm{Cosine}72^\circ }$ $\to \mathrm{Risk\,Reality\,Phenomenon\,Phi} \varphi ={R}^{2}+\frac{2\mathrm{Cosine}72^\circ R}{AB}-\frac{{\left(2\mathrm{Cosine} 72^\circ \right)}^{2}}{AB}$ where line segment AB defines the stability measurement and *R* defines the risk reality (*z *scores) measurements.

The Risk Reality Phenomenon Circle originates from the interconnecting 27 arcs that continuously loop creating a circle. The study proved that the geometric center metatheory was not located at the centroid of the circle. The opposite line segment Vertex 20 defines the Risk Reality Phenomenon Upper Limit. The arcs connecting the Vertex 21, Vertex 22, and Vertex 23 point toward the Risk Reality Phenomenon Spiral Tail.

**Fig. 4:**
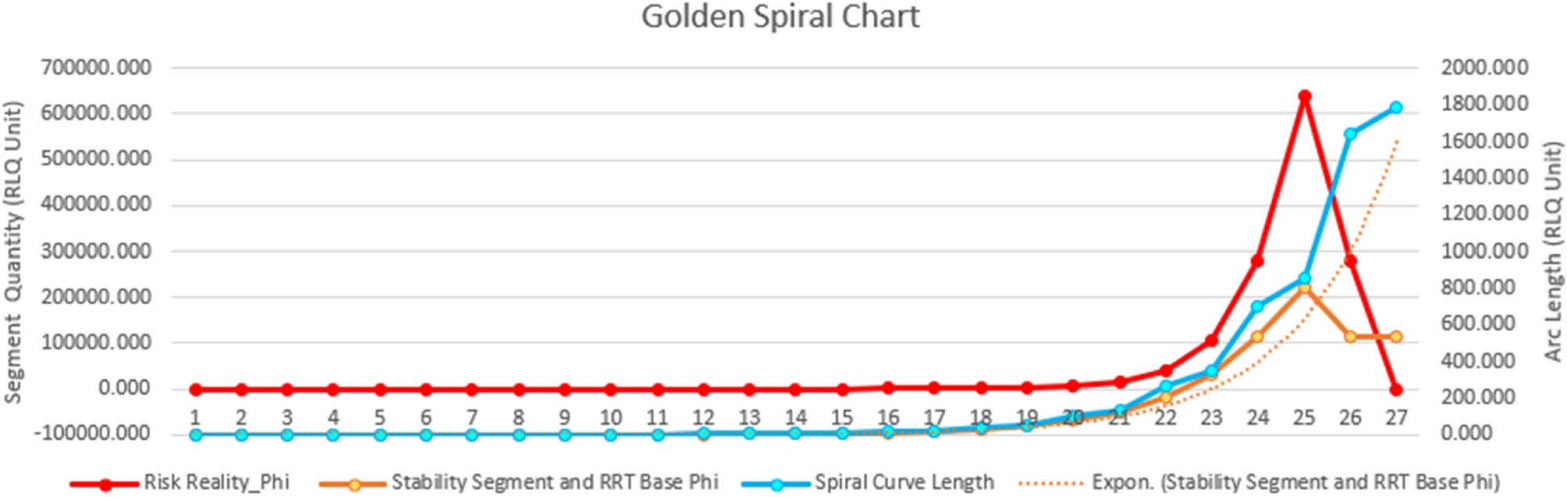
Risk reality phenomenon Phi chart [1, 2]

Figure 4 disclosed the relationships of the geometric center metatheory and stability measurement when Risk Reality Phenomenon Phi is assessed equal to one quantity unit. Similarly, Phi measurement at the upper limit and the spiral tail logged revealed where the inflection starts.

#### Vertices of resiliency

A Vertex of Resiliency metatheory refers to apex of the Risk Reality Phenomenon Triangle where the Sine 18° asymptotic segments that measures (assess) responsiveness and responsiveness, respectively. Both the responsiveness and responsiveness are reckoned at an angle of 36° opposite line segment symbolizing the risk measurement that varies from resilient (risk reality phenomenon measurement equal to 0.008 units), balanced (risk reality phenomenon measurement equal to 1-unit), and risk reality 1 < *z *scores > 125 risk *z *scores.

#### DRRM-circumspectial isometric stages grounded by risk reality phenomenon Phi

The Risk Reality Phenomenon Phi Circle creates some knowledge in which the asymptotic line segments were drawn connecting the Vertex Segments 23, Vertex 24, and Vertex 25. These 3 vertices divide the circle into three parts. By connecting these three vertices to the geometric center (0,0) it creates the reference lines that define the State of Prevention, State Preparedness, and Disaster Phase. To complete the DRRM Cycle, two horizontal lines were drawn that both passed through the geometric center. As these lines intersect the Risk Reality Phenomenon Circle it creates the State of Emergency and Post Disaster Phase [1].

**Fig. 5:**
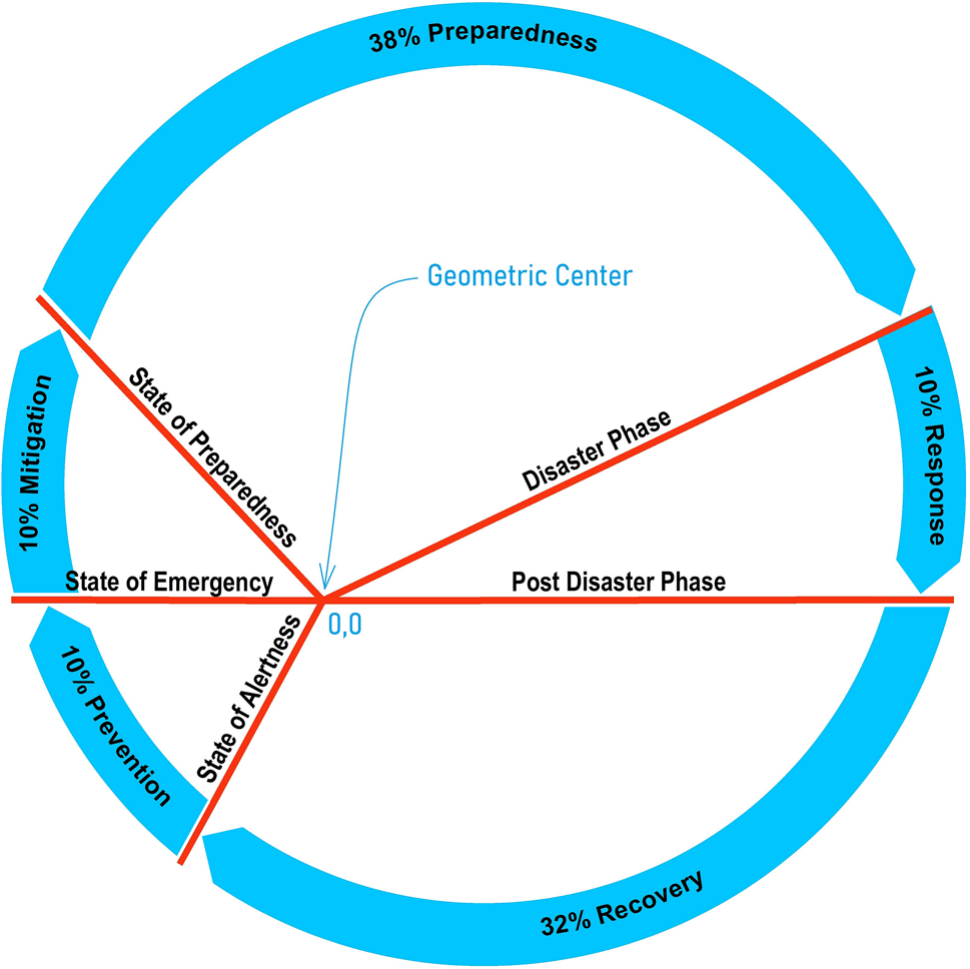
Risk reality phenomenon Phi cycle [1, 2]

Figure 5 shows the red lines which act as the partitions when risk reality phenomenon Phi are reckoned in the geometric center (0,0). These five partitions are represented by light blue arrows shows where the risk reduction and management begins: Prevention assessment starts at the State of Alertness and ends as State of Emergency, Mitigation assessment starts at the State of Emergency and ends when Preparedness Phase starts, Preparedness assessment starts at the State of Preparedness and ends when a calamity or disaster event starts, Response assessment starts where a calamity or disaster event stopped, and lastly Recovery assessment starts as soon Response Phase is over and end at the State of Alertness [1, 16].

The figure give clues on preparedness measurement will begin at the starting point where it joins the State of Alertness line if a do-nothing (0-prevention and 0-mitigation) scenario has been opted for the current situation. The red lines comprise as the reduction and management cycle regarded as partitions in the Risk Reality Phenomenon Phi Cycle in this study. Likewise, the Phi cycle hints at the combined 10% prevention, 10% mitigation and 38% preparedness needs are essential to get prepared. Similarly, it brings the knowledge on recovery is imaginable in a shorter response phase. Also, it hints at a longer recovery if a 0-prevention and 0-mitigation has been opted. Consistent with the fact that increasing residual risks from previous disasters added to the 0-prevention and 0-mitigation measurement and assumptions in risk assessment, the figure below suggests that full recovery is unlikely when preparedness needs are increasing, or preparedness is insufficient [1, 16]. This implies that the State of Preparedness is a measurement, where: preparedness is advanced when risk is near 0-risk quantity, preparedness is sufficient when risk is equal to one quantity and preparedness is insufficient when risk is greater than one quantity [1, 2, 15, 16]. Thus, when preparedness is sufficient it indicates that the State of Balance (stability) is likely [1, 2, 15, 16].

## Methods

### Organizing primary spatial data

Primary data were collected from OpenStreetMap and spatial data that were published and made available on the internet by government agencies. The input data were classified into an ordinal scale and depicted in a vector data formal language supported by the database of the GIS software used in characterizing risk elements’ features. In processing and quantifying the risk reality phenomenon as well as selecting stability criteria: safe space, comfortable host environment, and accessible site for settlement/resettlements as shown in Table 2 [1].

**Table 2:** Risk reality binning parameters

Risk reality binning parameters	Category				
Elements of risk reality	Very high risk (95–99% Hotspot)	High risk (90–95% Hotspot)	Moderate (Neutral)	Low risk (95–99% Coldspot)	Very low Risk (95–99% Coldspot)
Single to multiple hazards	≥ 500	≥ 450 < 500	≥ 180 < 450	≥ 60 < 180	< 60
	Very High Score	Score	Score	Score	Score
Landscape vulnerability	≥ 13 Critical environ	≥ 10 < 13 Somewhat Unsafe Environ	≥ 8 < 10	≥ 5 < 8 Somewhat stable environ	< 5 Somewhat stable environ
Passive exposure	5 Critical location	4 Unsafe Location	3	2 Somewhat stable location	1 Stable location
Preparedness based on highest-best-land use	5 Unprepared	4 Insufficient Preparedness	3	2 Somewhat Prepared	1 Advance in preparation
Competency	5 Incompetent	4 Somewhat Incompetent	3	2 Competent	1 Sustained competency
Coping capacity	5 In Bad Condition	4 Poor coping capacity	3	2 Somewhat high coping capacity	1 High coping capacity

The risk reality phenomenon was regarded as the geographical locations or hexagonal bins (host environs) where hotspots are possible and likely. It was assumed that multiple hazards are variating according to the following land conditions, these are: unstable slopes, critical elevations, and regularly flooded areas were given weights of 100; soil erosion-prone areas (based on soil characteristics) and areas near riparian rivers were given weights of 20; foreshore areas altered by storm surges which were given weights of 20, and areas susceptible to geomorphological changes caused by lahar and areas susceptible to lava were given weights equal to 100. The OSM building and road coincidence constitute the passive exposure and assigned with the highest score. To extract exposure measurement, line objects (OSM road centerlines) and polygon objects (OSM buildings) were buffered with 100 m to extract nearness measurement and density using the Geoprocessing Mapping tools in ArcGIS [1–2].

Table 2 shows the aggregated risk element’s scores which were classified into five classes: very high to extremely high (95%–99% risk hotspot information), high (90%–95% risk hotspot information), moderate (Neutral), low (−90%–95% risk coldspot information), and very low to absent (−95% to −99% risk coldspot information) [1–2, 15–16].

### Risk reality phenomenon assessment

The study was designed to contextualize and examine risk hotspot and coldspot information to analyze what suffered by the National Housing Authority (NHA) resettlement sites in Albay. The GIS overlay operations and hexagonal binning techniques were applied to store and sort risk reality phenomenon measurement. The researcher assessed the spatial patterns and quantified the risk elements’ statistical data to determine where the risk hotspot and coldspot information using the ArcGIS’s Getis-Ord Gi* statistics and Moran’s *I* test tools. The following methods were undertaken enhanced resettlement site selection modeling, review of the risk reality phenomenon measurement with the site selection model parameters for settlement or resettlement sites [1].

#### Hexagonal binning technique

**Fig. 6:**
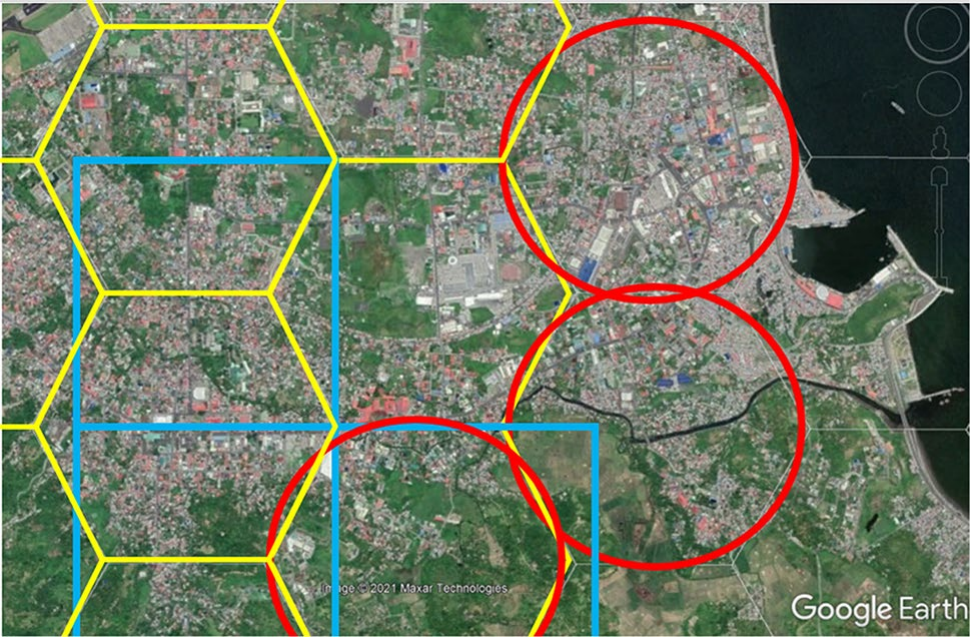
Tessellated Hexagonal Bins (Legazpi City, Albay)

The risk hotspots and cold spots binned information within the 25 km range from the crater of Mayon Volcano in Albay were analyzed to find where the development trends (and sprawl) and constraints (risk hotspots) are. The 1,541 hexagonal bins were created in ArcGIS platform comprising the honeycomb-like hexagonal polygons that were arranged perfectly to store and sort the input elements of risk measurements. Each bin measures 100 Ha in which it plays an important role in analyzing the spatial variation of risk reality measurements in Albay. Each bin depicts the degree of confidence and rendering of risk hotspot or cold spot information. Visualization of the Risk Reality Phenomenon on this basis (real physical space) is possible using hexagonal bins. The hexagonal bin segments comprise a 500-m distance in the real world wherein an average person with a 50-cm pace factor can walk will take 1,000 steps within 3-min at a 3-m/s speed to traverse one side of a hexagon. To conduct field verification, it will take at least 18 min to walk around the flat environment. Figure 6 shows that hexagonal bins are a better way to represent data than square grids [1–2].

#### Situational analyses

The upper portion of Mayon Volcano is a natural park (located within the permanent danger zone) reserved for the conservation of native plants and animals, their associated habitats, and cultural diversity. A 24.4 km road network provides access to the 8 km extended danger zone (for production forest and agricultural uses). Without seeing the force of sprawl as people develop their lands and construct buildings near the gullies, alluvial fans at the foot slopes of the volcano, and old railroads are ruined by repetitive volcanic eruptions. People take on disaster risk as multiple hazard events that can occur repeatedly. The undesired buildings were built along rights-of-ways, such as barangay/purok roads, farm-to-market roads, trails, and tracks of trucks hauling gravel and sand needed to develop cities and municipalities, dikes, evacuation routes, and so on. The barangay (village) nodal centers and the locations of the barangay halls are interconnected with road and road-like patterns. The density of buildings surrounding the barangay nodal centers varies depending on the topography and other landscape features, such as river and bank erosion due to severe rainfall [1–2].

The Risk Reality Phenomenon Phi enhanced resettlement site selection modeling in ArcGIS platform mimicked the Risk Reality Phenomenon in Albay. The Risk Reality Phenomenon Phi revealed hexagonal binning technique is useful in risk assessment using the Getis Ord Gi* (geostatistical tool). The Moran’s index was applied to characterize the seven levels of significance of risk hotspot information in ArcGIS platform [1–2].

#### Risk-areal differentiation

In this study, risk areal differentiation refers to the method used to obtain the difference between two calculated risk quantities, wherein negative residual values imply worse risk reduction actions are applied to reduce risk. In contrast, positive residual values affirm corrective risk reduction measures. The geographic locations (point features) of the 25 resettlement sites were overlaid with hexagonal bins (polygon features) using ArcGIS’s Geoprocessing Tool. The intersection of point and polygon features made it possible to transfer the hotspot, random and cold spot *z *score attributes from polygon to point map features. Then, the areal differential technique was applied to obtain the residual risk (Δ*R*) [1].

#### Risk reality phenomenon assessment

**Fig. 7:**
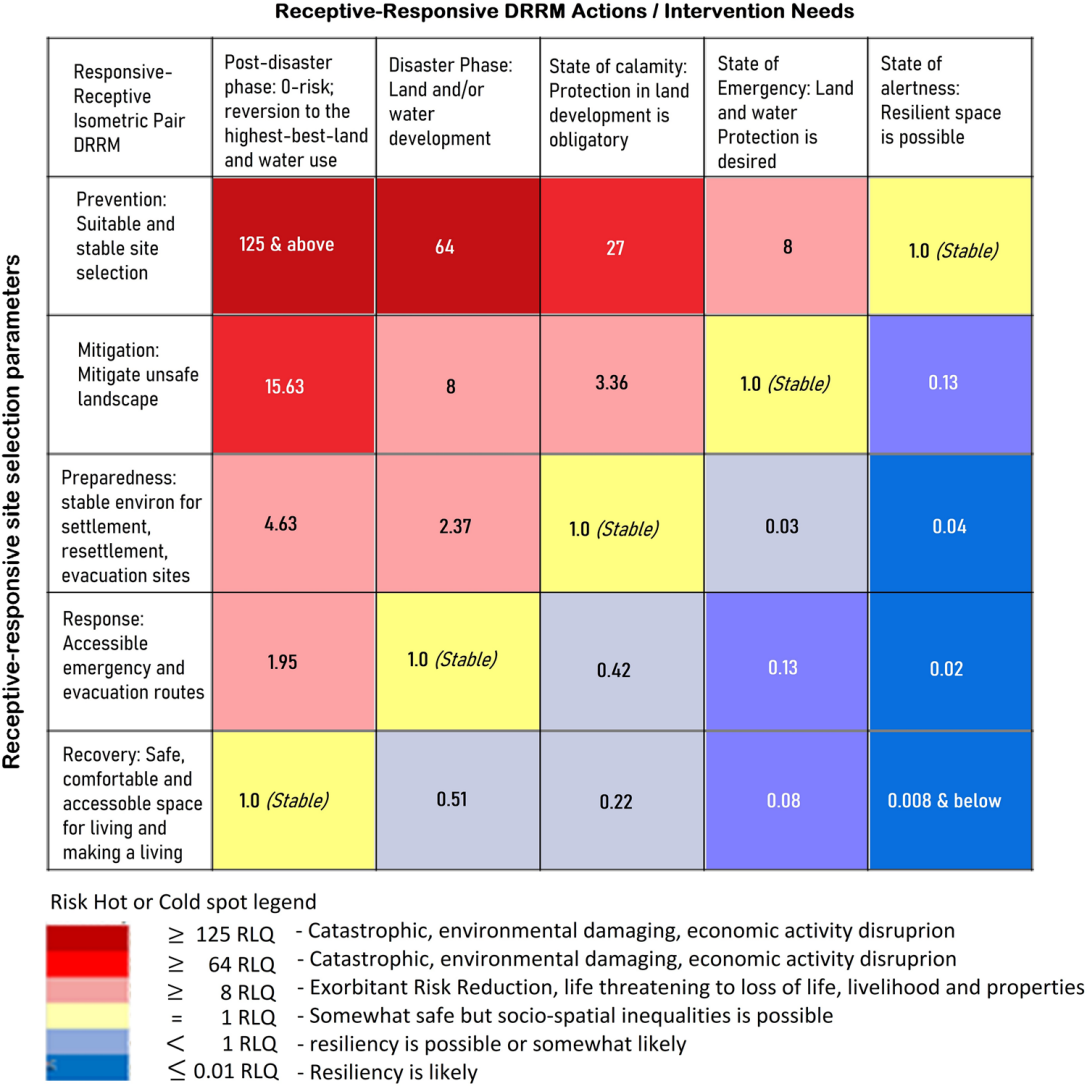
Receptive-Responsive DRR Isometric Index

The Receptive-Responsive Disaster Risk Reduction Isometric Index, as shown in Fig. 7, was created to check the stability in the host environment where the resettlement sites are located. It is useful to check the host environment based on stability criteria: safe, comfort, and accessibility. A resettlement may be safe but not necessarily comfortable and accessible or that may be comfortable temporarily (somewhat distant) but not safe or resilient. Receptive-Responsive Disaster Risk Reduction Isometric Index draws a partition that was based on *Metatheorems* which created the Risk Reality Phenomenon Phi Cycle as shown in Fig. 5. Figure 7 shows the imaginary line that connects the five cells which act as the partition when risk reality phenomenon Phi is less than or greater than one unit. It was used to assess the stability or instability of the 25 resettlement sites. The DRRM cycle: Prevention, Mitigation, Preparedness, Response, and Recovery were isometrically paired with the DRRM Circumspecial stages: State of Alertness, State of Emergency, State of Preparedness (status), Post Disaster Phase (Response), and Post Disaster Phase (Recovery) to find where the risk is equivalent to one unit to draw the risk from resiliency. Figure 7 disclosed the Risk Location Quotients (RLQ) derived from the pairing of the DRRM cycle and circumspectial stages. A catastrophic and environmental damaging or economic activity disruptions in terms of RLQ ranges 64–125 risk quantity. Exorbitant risk reduction or life-threatening, and loss of property or livelihood ranges from 8 to 64 risk quantities. The RLQ equal to one unit risk quantity constitutes a somewhat safe host environment but socio-spatial unfairness is possible. An RLQ quantified as less than one unit of risk implies resilience is somewhat likely. Resilient based on the Receptive-Responsive Disaster Risk Reduction Isometric Index begins at 0.01 RLQ or lower [1–2, 15–16].

**Fig. 8:**
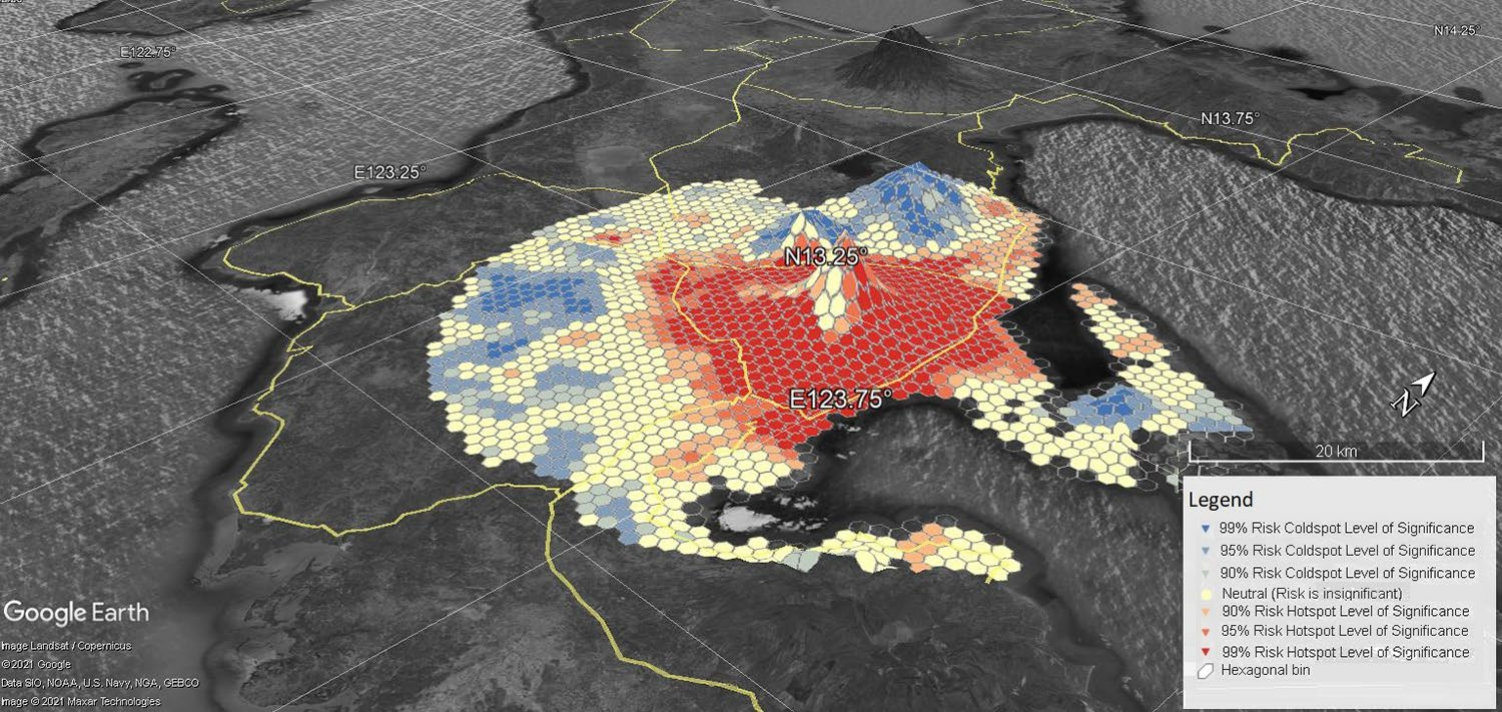
Risk Hotspot and Coldspot Binned Information in Google Earth Platform

Figure 8 shows the risk knowledge that implies the more people (represented by building footprints that are binned in the hexagonal polygons in ArcGIS platform) utilize danger zones, the more financial resources are required to maintain the 0-casualty goal of Albay. The Risk Hotspot and Coldspot Binned Information in Google Earth Platform disclosed the binned risk hotspot or coldspot information in terms of z-scores are as follows: 294 hexagonal bins or 2,900 hectares of land are labeled as significant hotspots at the 99% level, 71 hexagonal bins are labeled significant hotspots at the 95% level, 31 hexagonal bins are significant hotspots at the 90% level, 113 hexagonal bins are significant cold spots at the 90% level, 185 hexagonal bins are significant cold spots at the 95% level, and 97 hexagonal bins are significant cold spots at the 99% level. The ArcGIS’s Getis-Ord Gi* Statistical and Moran’s I Test results also identified general hotspots in areas with z-scores varying from 1.65 to 1.96, covering the following resettlement sites: Legazpi City, Ligao City, Tabaco City, Bacacay, Camalig, Daraga, Guinobatan, Libon, Malilipot, Malinao, Oas, Polangui, Sto. Domingo and Tiwi. In contrast, the cold spots are generally depicted by *z *scores between < 1 and *z *scores ≤ 2.58, which cover the following watershed divides: Balobo (Ligao-Guinobatan area), Banwang Gurang (Camalig-Jovellar area), Ogod (Camalig-Jovellar area), Polangui (Polangui-Oas area), Quinali ‘A’ (Camalig, Guinobatan, Ligao, Oas and Polangui area), Quinali ‘B’ (Malinao-Tabaco), Taque and Tiwi-Sangay area. Other fields are barely random values [1–2].

**Fig. 9:**
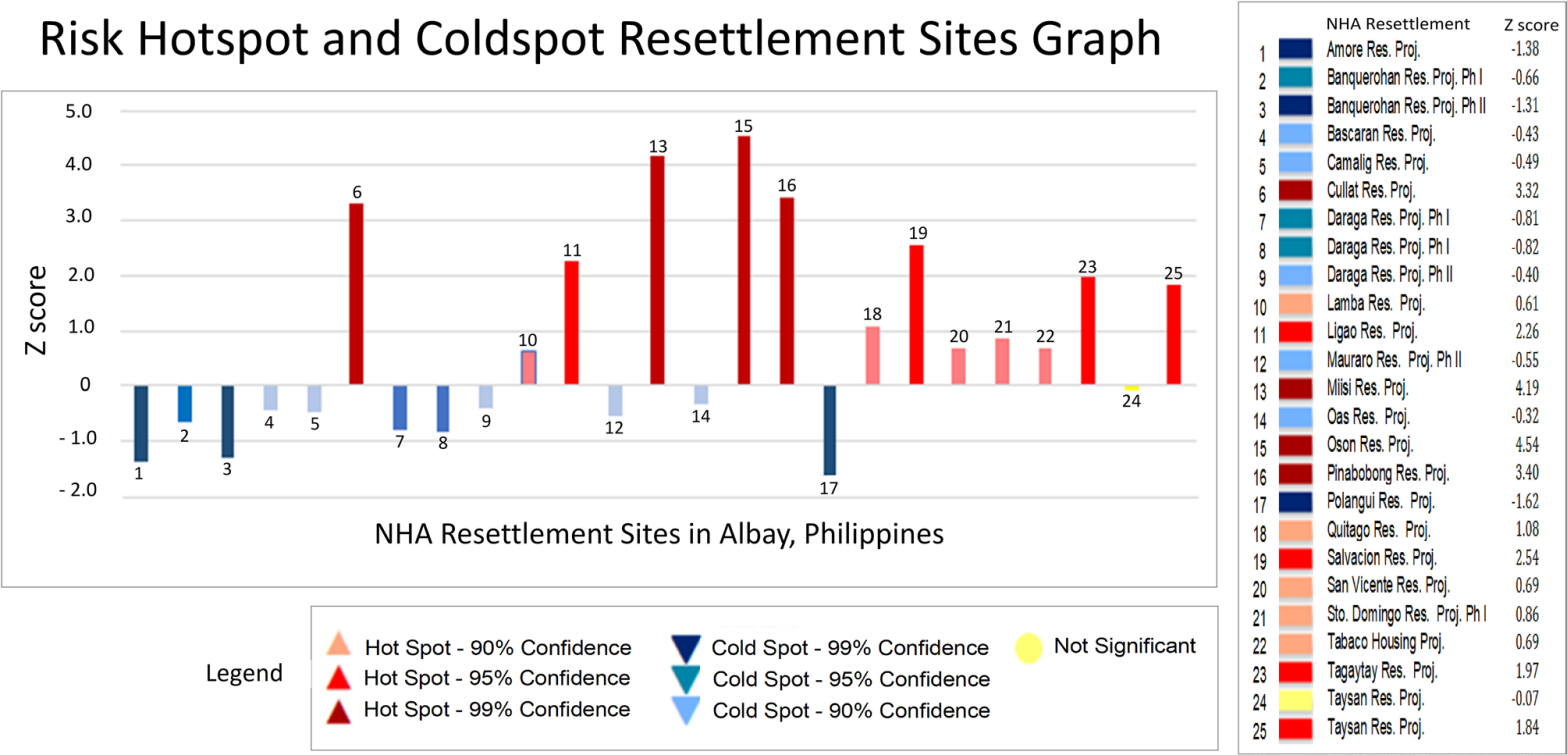
Stability Measurements in Resettlement Sites in Albay, Philippines

## Results

### Risk reality phenomenon assessment findings

The exposed residents (population displaced) who were relocated to the 25 National Housing Agency Resettlement Sites in Albay showed the significance of selecting the location of the host environment with the following site selection criteria, these are safe, comfort, and accessibility. The study disclosed the 14 out of 25 resettlement sites (host environs) that are in bad land conditions will continue to suffer if the government delays relocating the exposed residents to safer, comfortable, and accessible host environments. These findings constitute the risk realities suffered by the 14 resettlement sites. Stability in resettlement sites is assessed in terms of receptive or responsive risk metatheory using the Receptive-Responsive DRR Isometric Index as shown in Fig. 7. In contrast, instability in the host environment is assessed as a risk reality phenomenon suffered by resettlement sites using the Risk-Areal Differentiation principle. *N* indicates no suffering based on the Phi (scientific data) findings, although some recipients of housing projects may disagree with these geographical information modeling results. The *z *scores determine the spatial characteristics of the host environment’s land condition and its proximity to danger zones, and the social aspect is interpreted as the comfort in accessing the residents’ source of income and basic socioeconomic support services. Table 3 shows the risk reality phenomenon assessment result disclosing what the host environment suffered in terms of hotspot or coldspot *z *scores [1].

**Table 3:** Risk Reality Phenomenon Phi assessment result in resettlement sites, Albay

ID	Resettlement sites	Risk reality phenomenon phi						Stability measurement		
		Present (Year 2020)			Future					
		*Z *score	Type	Residual	Phi	Impact	Trend	Receptiveness	Responsiveness	Risk Suffered
1	Amore resettlement project	−1.38	C	N	N.A	N	C	Li	Li	N
2	Banquerohan resettlement project Ph I	−0.66	C	N	N.A	N	C	Li	Li	N
3	Banquerohan resettlement project Ph II	−1.31	C	N	N.A	N	C	Li	Li	N
4	Bascaran resettlement project	−0.43	C	N	N.A	N	C	Li	Li	N
5	Camalig resettlement project	−0.49	C	N	N.A	N	C	Li	Li	N
6	Cullat resettlement project	3.32	H	Y	11.02	Y	F	U	U	Y
7	Daraga resettlement project Ph I	−0.81	C	N	N.A	N	C	Li	Li	N
8	Daraga resettlement project Ph I	−0.82	C	N	N.A	N	C	Li	Li	N
9	Daraga resettlement project Ph II	−0.4	C	N	N.A	N	C	Li	Li	N
10	Lamba resettlement project	0.61	H	Y	0.37	Y	H	U	SLi	Y
11	Ligao resettlement project	2.26	H	Y	5.12	N	F	U	SLi	Y
12	Mauraro resettlement project Ph II	−0.55	C	N	N.A	N	C	Li	Li	N
13	Miisi resettlement project	4.19	H	Y	17.53	N	F	U	U	Y
14	Oas resettlement project	−0.32	C	N	N.A	N	C	Li	Li	N
15	Oson resettlement project	4.54	H	Y	20.61	Y	F	U	U	Y
16	Pinabobong resettlement project	3.4	H	Y	11.57	N	F	U	U	Y
17	Polangui resettlement project	−1.62	C	N	N.A	N	C	Li	Li	N
18	Quitago resettlement project	1.08	H	Y	1.18	N	C	U	SLi	Y
19	Salvacion resettlement project	2.54	H	Y	6.44	Y	F	U	SLi	Y
20	San Vicente resettlement project	0.69	H	Y	0.48	Y	H	U	SLi	Y
21	Sto. Domingo resettlement project Ph I	0.86	H	Y	0.74	Y	H	U	SLi	Y
22	Tabaco Housing Project	0.69	H	Y	0.48	Y	H	U	SLi	Y
23	Tagaytay resettlement project	1.97	H	Y	3.89	Y	H	U	SLi	Y
24	Taysan resettlement project	−0.07	Ra	Y	N.A	N	Ra	U	Li	Y
25	Taysan resettlement project	1.84	H	Y	3.4	Y	H	U	SLi	Y

*C Coldspot; H Hotspot; Ra → Random; F Fuzzy Reality; Y Yes; N No; VH Very High; H High; M Moderate; L Low; VL Very Low; N.A. Not Applicable; Li → Likely; SLi → Somewhat Likely; U → Unlikely*

### Risk reality phenomenon in resettlement sites in Albay

Based on the z-scores in Table 3, the following host environment suffered risk remainders: Cullat (Daraga), Lamba (Legazpi), Ligao City, Miisi (Daraga), Oson (Tabaco City), Pinabobong (Tabaco City), Quitago (Guinobatan), Salvacion (Tabaco City), San Vicente (Tabaco), Sto. Domingo, Tabaco Housing Project, Tagaytay (Camalig), and Taysan (Legazpi City). The result disclosed that the z-scores and hotspot and coldspot classification are variating: very low indicates a significant cold spot at the 95%–99% level or very low-risk class (VL); low indicates a significant cold spot at the 90% level or low-risk class (L); moderate indicates random negative or positive *z *score values between −89 and 89% confidence (M); high indicates a significant hotspot at the 90%–95% level or high-risk class (H), and rising uncertainties indicate a significant hotspot at the 99% level (VH).

The risk spreading in the 14-host environment (resettlement site) creates the knowledge that 4 host environments significantly need attention, these are Oson and Pinabobong in Tabaco City and Cullat and Miisi in Daraga town. Of these four sites, Cullat is a unique case in which it carries the risk reality phenomenon Phi of the hexagonal bin hosting it. This constitutes the limitations of using small scale maps as it generalizes some important details about the landforms of the host environment. Consequently, the Cullat resettlement site carries a higher *z* score. This study acknowledges the limitations of the data model, which is accurate for 1:50,000 scale input maps. This modeling is suitable for macro planning and rapid risk assessment. But the same approach to select suitable and stable sites for resettlement projects may be adopted for micro-planning or comprehensive risk assessment [1].

### Analysis and visualization of risk reality phenomenon in resettlement sites

**Fig. 10:**
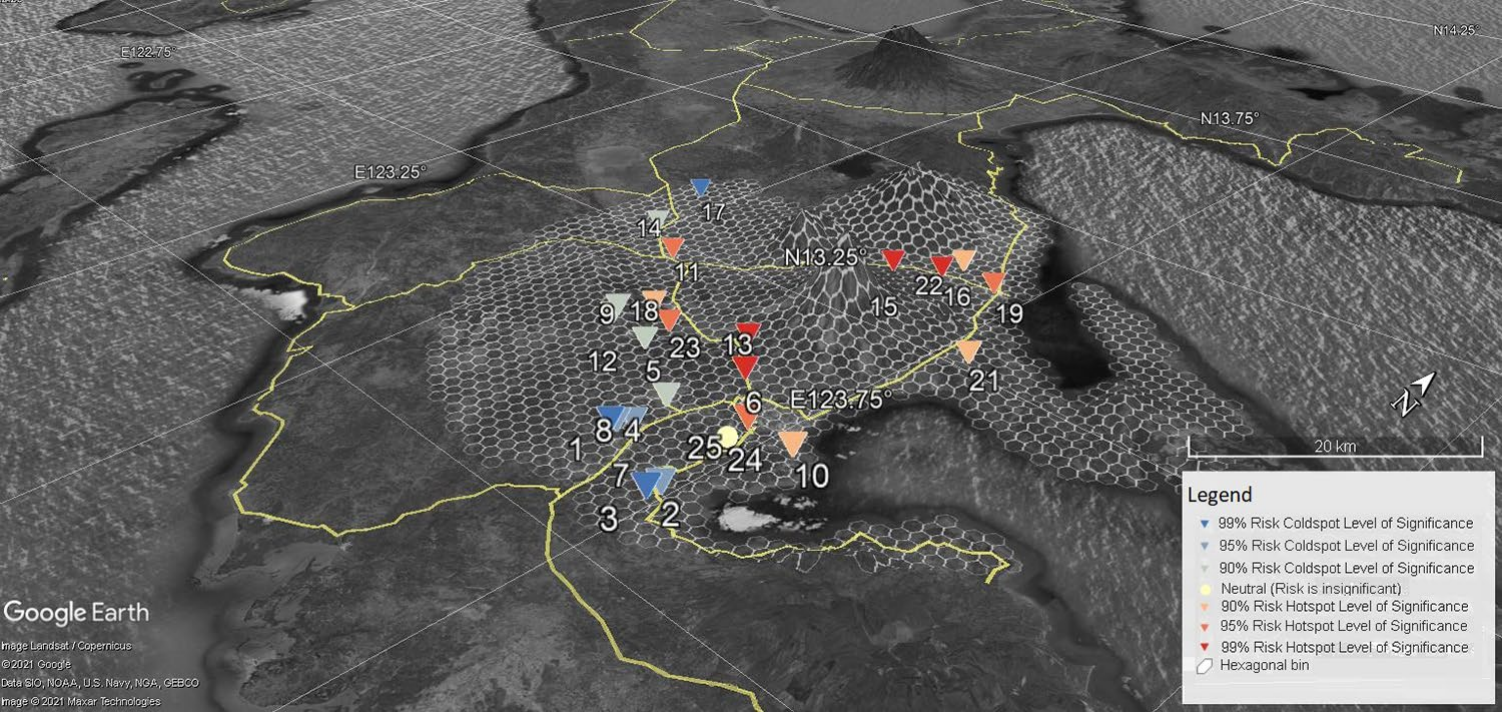
Risk Reality Phenomenon in NHA Resettlement Sites in Albay, Philippines

The Risk Reality Phenomenon measurements in NHA Resettlement Sites were overlaid with the risk hotspots and coldspots statistical to extract and carry the risk information of the hexagonal bin where it intersects with. It is presented in Google Earth Platform as shown in Figs. 8 and 10 to visualize the distribution of risk hotspot or coldspot host environment. Figure 10 creates a risk knowledge about the nearness of the resettlement sites to the foot slopes of the Mayon Volcano the higher consequences to be situated in a risk hotspot environment. The farther the resettlement sites the safer but not necessarily imply it offers comfort to relocated residents due to travel distance to the place of employment or livelihood or to get basic social services often located in urbanized areas as shown in Fig. 10 [1].

## Discussions

This section explains the significance of examining the 25 resettlement sites as samples of host environment information that depict the risk reality phenomenon Phi that hints at 0-risk when exposure (as an independent variable of risk) is nil. Nil exposure information creates risk knowledge on risk residual after measuring the host environment of unsettlement (land natural condition of the host environment) and resettlement (to new built environment) sites. However, these risk knowledge needs further study because the other three elements of risk that comprise the overall capability: preparedness, competency and coping capacity are limited to income level classification of the local government which were distributed equally stored and sorted in all the hexagonal bins covering the 25 km range reckoned from the crater of the volcano. The risk reality phenomenon information of respective resettlement sites carries the binned risk information where it coincides. The results disclosed the 14 out of 25 National Housing Authority Resettlement Projects in Albay creates a risk knowledge that those sites near or within the 10–15 km range measured from the crater of Mayon Volcano are likely remained at risk to volcanic related hazards compared with other sites. In contrast, those sites outside the 15 km range are possibly resilient. The risk knowledge originated from the risk information based on the Fibonacci spiral again creates knowledge on improper land utilization. The 14 resettlement sites which remain at risk signify that it does not conform with the best-highest-best land use creates the land use sensitivity is relevant to risk assessment because it reveals a way to mainstream risk information into land use plans and zoning and weigh bearable risk that is acceptable and adaptable at all levels [1, 9].

### Risk reality phenomenon

Consistent with the philosophical theories on risk reality phenomenon, the risk hotspot or coldspot assessment were based on the resettlement (stability) site selection criteria: safe, comfort and accessibility. The social stability and risk reality phenomenon assessment in the National Housing Authority Resettlement sites case study was done to generate risk knowledge [1, 8–9]. GIS modeling mimicked the 25 resettlement sites (samples) which proved that the risk reality phenomenon is measurable that creates the risk knowledge on the 14 sites which need land-use replanning. In land-use planning or site selection process, we must understand that the landforms naturally endanger residents' lives, in contrast resiliency arises when exposure is eliminated. When we dare to prolong people’s vulnerability from the extrinsic effects of bad landform condition of the host environment (stored and sorted in hexagonal bins), we are allowing them to get exposed [1, 9].

The risk knowledge originated from the risk reality and trend metatheories are essential in assessing the hierarchical relationship between highest-best-land use and preparedness insufficiencies worsened by unprevented (analogous to unreceptive risk reduction actions) and unmitigated (analogous to unresponsive risk reduction actions) risks. It disclosed complications in the State of Preparedness as shown in Fig. 5 can worsen the coping mechanism of the displaced or resettled people [1–2, 9]. The author agrees with Uy et al (2011) that vulnerable communities in Albay faced increasing threats to livelihood and safety and understanding the land conditions is indispensable in planning and formulation of appropriate local adaptation strategies and actions at local level [3–4, 7]. The author agrees also with Owen et al (2020) and Aven and Flage (2018) that risk hotspot information incorporates the risk knowledge into a transparent framework to incorporate the cost of extrinsic effects of risk to keep stability and to sustain the resettlement projects [1–3, 8–9]. The risk reality phenomenon in this work originated from the metatheorem on the vertex of resiliency where it rests at the Sine 18° angle of the isosceles triangle where the opposite segment denoting the stability has a length of one unit of risk based which originated from golden ratio and metatheorems of this study advances risk assessment [1]. The Risk Reality Phenomenon Phi that is trending was based on the Fibonacci Golden Ratio and Schoen Golden Triangles, mathematically written as Phi *φ* = *R*^2^ + 0.00494427*R *− 0.00305572 where Risk Reality (R) is contextualized in this study as the function hazards, landscape vulnerability of the host environment, passive (unchanging geographical location) exposure, preparedness level sufficiency, level of competency of the government to withstand the effects of natural calamities or disasters, and coping capacity of the affected people) [1].

### Risk knowledge on nil exposure

**Fig. 11:**
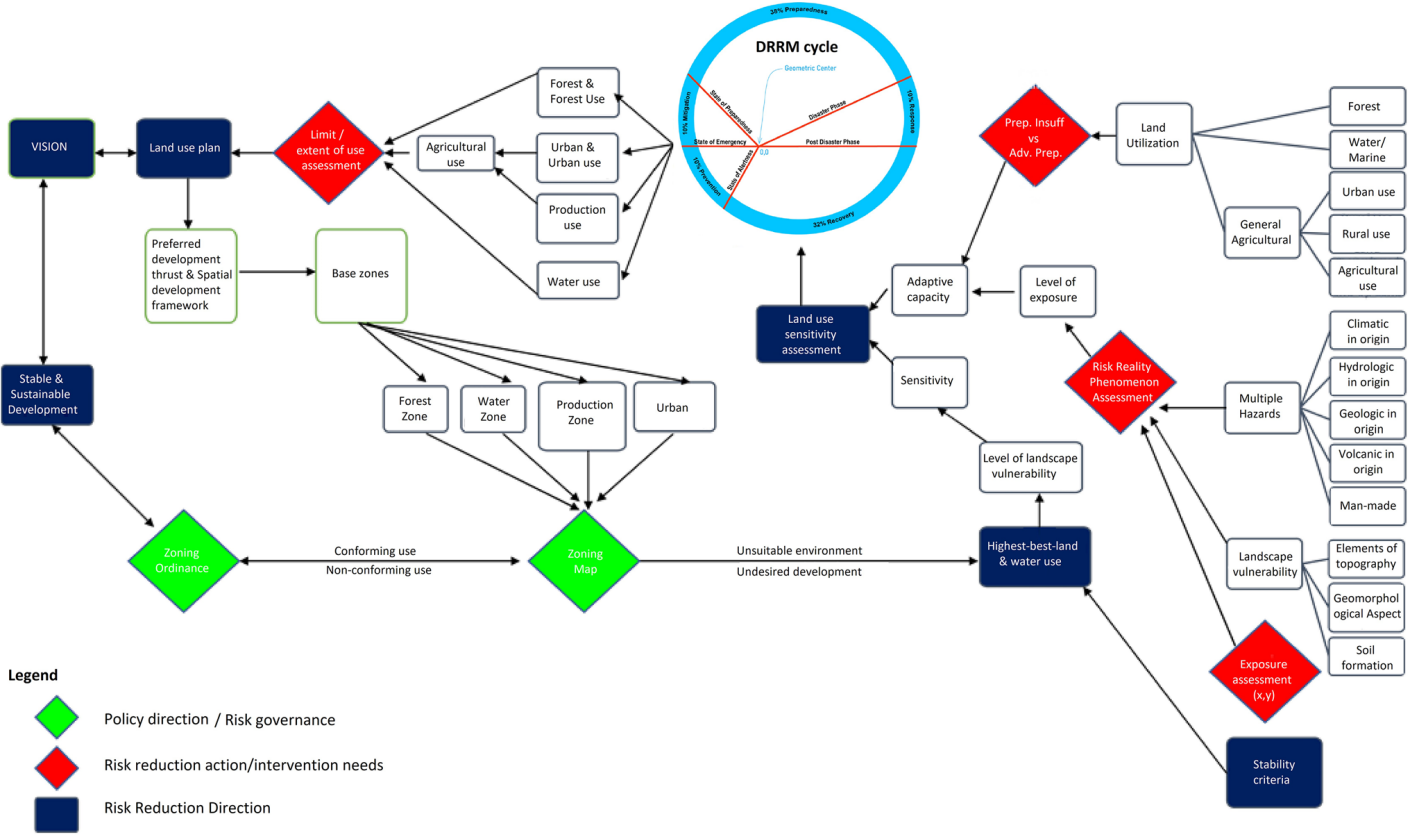
Land use sensitive, balanced and sustainable development Hierarchical relationships

When people are steadily allowed to occupy danger zones or risk hotspots areas it hints at pressing potential disaster. The fact that passive exposure is involved as an independent variable when paired with coping capacity hinted at the geophilosophical location as a conceptual space as the most important development control related to land utilization and zoning the resettlement site (built environment) as well as the urban expansion areas. In selecting a resettlement site, stability selection parameters urge planners, decision-makers, and development managers to select the most reasonable host environment where to resettle or relocate the displaced residents. The thought-provoking point resulting from this research is eliminating the exposure (unchanging geographical location) variable of disaster to revert the undesired developments to its highest-best-land use to realize a balanced and sustainable development. Nil exposure implies near zero-risk described as resiliency that is attributed to the highest-best-land use. It can be interpreted as a resettlement or relocation planning scenario where 0-risk can be assumed in unsettling the residents. A 0-risk or near-zero policy is ideal although it is difficult to evade passive exposure [1–2, 15–16]. The author agrees with Hofmann (2007) that there is no clear definition on the concept of “resilience” even though this term is widely used in the research. She also agrees with him that resilience (0-risk) concept depends on the situation (actual land utilization) before and the situation after the disturbance (hazard events) occurred [1, 6] Fig. 11 shows the hierarchical relationship between highest-best-land use as a scientifically informed policy direction and preparedness sufficiency which benchmark what, when, and where to start balancing the current and future growth of cities and municipalities.

### Land use sensitivity risk knowledge

Although Albay successfully achieved a zero-casualty goal for the past two decades under a strict communication protocol it remained at risk [11]. The author agrees with Espinas (2013) that land conditions of the host environment and risk reality phenomena should not hinder development [12]. The author also agrees with Cuevas et al. (2015) that there is limited information to investigate the land use gaps (sensitivity) to adapt to changing climate.[13] The land use sensitivity assessment (as an action to take is reliant on (binned) information on the level of exposure (extent of land use that exposes residents) and the level of landscape vulnerability (host environ) which link the sensitivity with the highest-best land use (development growth direction) based on stability and resiliency parameters [1, 15]. Land use sensitivity is reliant on adaptive capacity of the exposed elements relative to their vulnerability that are extrinsic to the host environment regarded as vulnerable landscape or landform that rule what are the highest-best land uses based on safe, comfortable, and accessible site parameters [1, 15–16]. Mainstreaming the land use sensitivity (understood as the space utilization that varies from: no-build zone to low unsuitable host environment or somewhat unsafe but uncomfortable area, protecting the remaining resilient sites suitable for forest and water zones, and balance utilization of the production areas or agricultural and settlement) into the land use plans and zoning ordinances are development control processes useful to determine the conforming or non-conforming land uses or areas that are risk hotspots [1–2, 11–12, 14–16].

When planners, engineers, and developers fail to see the land use sensitivity as an external factor of the host environment (bad landscape condition) then intrinsic negative effects to the resettlement projects and residents are likely. Changing landscape conditions of the host environment and preparedness insufficiency (Abante 2018) can worsen the coping capacity of the displaced people. As the risk location information varies the risk computing at the household level becomes crucial in disaster risk reduction to meet the targets and priorities for resiliency action plans as stated in the Sendai Framework. The author agrees with Barua et al (2020) that risk-sensitive land use planning is an action that has been considered important in recent years as it mainstreams disaster risk reduction and management parameters into land-use plans [1, 12–16].

#### Stability criteria as scientifically informed policy direction

This study proposes to apply the risk knowledge created by the discovery of the stability criteria and borderline that partition the risk reality phenomenon phi from resiliency where risk is less than one or nearing zero. It originated from the risk reality phenomenon concept model where the vertex of resiliency rests at Sine 18° angle and opposite stability segment of an isosceles triangle having a length of one unit of risk based which originated from golden ratio and isosceles triangle. The 25 series of isosceles triangles are reckoned from 0,0 position (geometric center). It is on the geometric center where resiliency (near 0-risk or 0.008 units) rests and positioned at the 0.0 point. In contrast, risk reality is anywhere in the risk spiral that connects the geometric center up to its tail denoting where the risk reality is at the upper limit. The effect of extending the risk reality measured above the upper limit divulges that a circle is formed when the spiral tail returns to its original location [1].

The absence of stability criteria to properly identify and locate risk hotspots tend to increase undesired developments which relatively increases the number of exposed people, properties, critical socioeconomic support infrastructure, and so on. The stability criteria were a proven factor to mainstream risk measurements into land use plans and local resilience policies (in built environments) to build adaptive capacity from near-zero to extreme exposure which can also lead to less cost-effective risk reduction actions [1, 10]. The hexagonal binning technique proved that it could generate knowledge about risk hotspots and coldspots to keep stability and to sustain the resettlement projects or to adopt strategies that can lead to less cost-effective risk reduction actions [1–2, 10, 15–16]. The author agrees with Bosher and Chmutina (2017) that the consequence impacted built environment is correlated with instability that can create intrinsic vulnerabilities and active exposure of residents [1, 10]. She also agrees with (Owen et al. 2020) these consequences could lead to destruction of housing projects that can displace the residents or alteration of the host environment which influences the market (economy) [1, 3]. The State of Preparedness stance is where the stage where preparedness measurement begins and stops when a calamity or disaster event starts [1].

#### Scientifically informed DRRM cycle partitions

The geophilosophy on risk realness is akin to the DRRM cycle and circumspectial stages (DRRM partitions as shown in Fig. 5) to restore and sustain the resettlement projects in Albay [1, 16]. The discovery where the DRRM cycle creates the risk knowledge that getting prepared is comparable to fully recovering from the effects of recent and past disasters. It also suggests that the unprevented and unmitigated risks are added to preparedness needs which creates extraneous errors (common mistakes in assessing risk reality phenomena) that cause preparedness insufficiency. Ignoring the residual risk brought by previous disasters and extraneous errors in risk computing are likely to hinder stability or bearable risk or even block our ability to become resilient. Unlocking this risk metatheory on well-defined partitions of the DRRM cycle offers a better way to rethink how to build-back-better or restore the vulnerable host environment by reverting the undesired (sprawl) developments to its highest-best-land use [1, 14, 16].

The practical significance of the Risk Reality Phenomenon Phi Cycle as shown in Figs. 5 and 11 is dividing DRRM cycle into five parts, these are: 10% prevention impact starts at the State of Alertness and ends as the State of Emergency, 10% mitigation impact starts at the State of Emergency and ends when Preparedness Phase starts, 38% preparedness impact starts at the State of Preparedness and ends when a calamity or disaster event starts, 10% response impact starts, where a calamity or disaster event stopped, and 32% recovery impact starts as soon Response Phase is over and end at the State of Alertness can attain a meaningful risk reduction and recovery to restore and improve living conditions in resettlement communities. The Risk Reality Phenomenon Phi cycle can influence the other decision, direction, and action to achieve stability and resiliency in resettlement sites to attain a meaningful risk reduction and recovery to restore and improve living conditions in resettlement communities. The Risk Reality Phenomenon Phi Cycle creates risk knowledge about preparedness sufficiency. It decreases when disaster risk is unprevented and unmitigated which can implicate preparedness insufficiency. The preparedness insufficiency can worsen the coping mechanism of the displaced or resettled people. It hints at unprevented and unmitigated risk is transformed to preparedness needs causing the increasing preparedness insufficiencies which entails extensive recovery to restart the State of Balance (stability) [1, 16].

The discovery of the DRRM cycle partitions creates policy direction to get prepared and to fully recover from the effects of recent and past disasters. It also disclosed the unprevented and unmitigated risks are added to preparedness needs which creates extraneous errors (common mistakes in assessing risk reality phenomena) that increase preparedness insufficiency [1, 16]. This risk knowledge led to the conclusion that continuing to ignore the residual risk generated by previous disasters and extraneous errors in risk computing likely to hinder stability (or achieving a bearable risk) or even block our ability to become resilient. The practical implication of unlocking the well-defined partitions of the DRRM cycle defies conventional thinking to properly reduce disaster risk to build-back-better, thus realizing a balanced and sustainable development that is consistent with the Sendai Framework [1, 16].

## Conclusions

It is concluded that risk reality phenomenon is measurable which can be used as a basis to examine the consequences of not employing stability to get prepared with the following selection criteria: safe space, comfortable environment, and open accessibility to the host environment in risk assessment. While it is difficult to evade passive exposure, stability selection parameters urge planners, decision-makers, and development managers to attain the risk sensitive spatial development option for the host environment to apply a near 0-risk policy reduction in various landforms in addition to the existing 0-casualty goal of Provincial Government of Albay, Philippines.

The practical implications of risk assessment are on generating some knowledge on risk realness that open a new avenue for scientifically informed policy and risk governance to adjust prevention and mitigation measures to get prepared. Bringing together the risk reality phenomenon measurements with stability criteria, nil exposure, near 0-risk, land use sensitivity policy direction it will not only advance preparedness or meaningful risk reduction or but likely keep a stable and sustainable as cities and municipalities while passing the impact of active exposure to the COVID-19 pandemic that affect coping capacity of the Albayanos to a new norm after the pandemic. This new norm is regarded as a change from ‘post disaster phase’ to the ‘state of alertness’ where recovery from the impact of risk remainders from past natural calamities and disasters now combined with a man-made disaster that activated the exposure to pandemic that worsen the preparedness, competency, and coping capacity because of loss of livelihood or source of income of the people and long-term economic aftereffects of COVID-19 in the Philippines.

It is further concluded that the scientifically informed risk reduction policy directions that originated from the geophilosophical risk realities perspective and metatheorems, right decisions, and corrective and sensitive actions can stimulate a forward-thinking risk governance to get prepared from the worsening risk reality that is trending and results that depends on the direction and decisions during the critical turning point (end of COVID-19 pandemic) will surely involve costly receptive and responsive risk reduction actions at all levels.

## Data Availability

N. A
